# A comprehensive histomolecular characterization of meningioangiomatosis: Further evidence for a precursor neoplastic lesion

**DOI:** 10.1111/bpa.13259

**Published:** 2024-04-02

**Authors:** Arnault Tauziède‐Espariat, Julien Masliah‐Planchon, Philipp Sievers, Felix Sahm, Volodia Dangouloff‐Ros, Nathalie Boddaert, Lauren Hasty, Oumaima Aboubakr, Alice Métais, Fabrice Chrétien, Alexandre Roux, Johan Pallud, Thomas Blauwblomme, Kévin Beccaria, Franck Bourdeaut, Stéphanie Puget, Pascale Varlet

**Affiliations:** ^1^ Department of Neuropathology GHU Paris‐Psychiatrie et Neurosciences, Sainte‐Anne Hospital Paris France; ^2^ Institut Curie, Laboratory of Somatic Genetics, PMDT, Paris Sciences Lettres Research University Paris France; ^3^ Department of Neuropathology Institute of Pathology, University Hospital Heidelberg Heidelberg Germany; ^4^ Clinical Cooperation Unit Neuropathology, German Consortium for Translational Cancer Research (DKTK), German Cancer Research Center DKFZ Heidelberg Germany; ^5^ Pediatric Radiology Department Hôpital Necker Enfants Malades AP‐ HP France; ^6^ Université Paris Cité, UMR 1163, Institut Imagine and INSERM U1299 Paris France; ^7^ Université Paris Cité, Institute of Psychiatry and Neuroscience of Paris (IPNP), INSERM U1266, Ima‐Brain team Paris France; ^8^ Department of Neurosurgery GHU Paris‐Psychiatrie et Neurosciences, Sainte‐Anne Hospital Paris France; ^9^ Department of Pediatric Neurosurgery Necker Hospital, APHP, Université Paris Descartes Paris France; ^10^ SIREDO Center Care, Innovation, Research In Pediatric, Adolescent and Young Adult Oncology, Curie Institute and Paris Descartes University Paris France; ^11^ Department of Neurosurgery CHU Martinique Fort‐de‐France France

**Keywords:** DNA‐methylation, meningioangiomatosis, meningiomas, NF2

## Abstract

Meningioangiomatosis (MAM) remains a poorly understood lesion responsible for epileptic disease. In the past, MAM was primarily described in the context of neurofibromatosis type 2 before being mainly reported sporadically. Moreover, the malformative or tumoral nature is still debated. Because a subset of MAM are associated with meningiomas, some authors argue that MAM corresponds to an infiltration pattern of these tumors. For these reasons, MAM has not been added to the World Health Organization (WHO) Classification of Central Nervous System Tumors as a specific entity. In the present study, we characterized a series of pure MAM (*n* = 7) and MAM associated with meningiomas (*n* = 4) using histopathology, immunohistochemistry, genetic (fluorescent in situ and DNA sequencing analyses), and epigenetic (DNA‐methylation profiling) data. We evidenced two distinct morphological patterns: MAM with a fibroblastic‐like pattern having few lesional cells, and MAM with a more cellular pattern. A subset was associated with the genetic alterations previously reported in meningiomas (such as a *KMT2C* mutation and a hemizygous deletion of chromosome 22q including the *NF2* gene). The DNA‐methylation profile, using a t‐distributed stochastic neighbor embedding analysis, evidenced that MAM (pure or associated with meningiomas) clustered in a separate group from pediatric meningiomas. The present results seem to suggest that MAM represents a neoplastic lesion and encourage the further study of similar additional series so that it may be included in a future WHO classification.

## Graphical abstract

Clinical and molecular history of meningioangiomatosis.

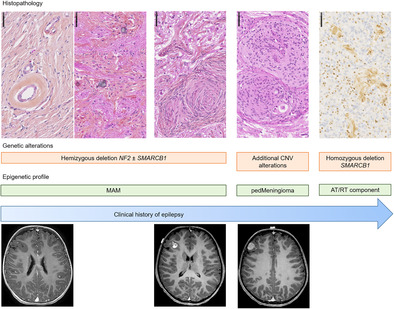

## 1 INTRODUCTION

Meningioangiomatosis (MAM) is a poorly studied, rare, benign, and epileptogenic brain meningovascular lesion. Only about 200 cases have been reported to date [1, 2]. The majority of MAM are sporadic, affecting predominantly male patients, younger than 20 years of age [3]. Epileptic seizures constitute the main symptom and represent a clinical concern with more than 80% of patients having uncontrolled seizures at the time of surgery [3]. Surgery is the first‐line treatment. Histopathologically, MAM is characterized by an intracortical meningovascular proliferation with a perivascular spread of spindle‐shaped cells along the Virchow‐Robin spaces. It often encompasses psammoma bodies, fibrosis, and white matter infiltration [3]. Because the malformative or neoplastic origin of MAM is still not well understood, this lesion is not part of the meningioma chapter of the current World Health Organization (WHO) Classification of Central Nervous System (CNS) Tumors. Very few genetic data for MAM are available in the literature. MAM may be isolated (pure) or associated with meningiomas (MAM + M), supporting the idea that MAM represents a potential tumoral pattern of infiltration. Moreover, for the majority of patients being treated by a complete resection and having epileptic seizure control, no recurrence at the end of follow‐up and no case of MAM transforming into meningioma has been reported. In this study, we performed histopathological and molecular analyses (including DNA‐methylation profiling) for 11 cases of MAM (seven pure and four associated with a meningioma) in order to further suitably characterize these lesions.

## 2 MATERIALS AND METHODS

### 2.1 Study design, patients, data collection

This study included patients diagnosed with MAM, provided by the consultation archive database (1993–2022) from the department of neuropathology at GHU‐Paris Psychiatry and Neurosciences, Sainte‐Anne Hospital. Epidemiological data (sex and age at diagnosis) and lesion and treatment‐related data (location of lesion, preoperative magnetic resonance imaging (MRI) features, duration of symptoms, extent of resection, relapses, and complementary treatments) were retrospectively analyzed. The extent of the initial resection was assessed by early (within 48 h) postoperative MRI. All patients' parents or legal guardians signed informed consent forms before treatment began. We obtained human subjects approval for genetic analyses.

### 2.2 Central histopathological review

The central pathology review was performed conjointly by two neuropathologists (ATE and PV). Samples were stained with hematoxylin‐phloxin‐saffron (HPS) according to standard protocol. For each case of MAM, the following pathological features were researched: cell density, pattern (nodules of lesional cells or only infiltration of Virchow Robin spaces), and psammoma bodies. Mitotic count was monitored using 5 high‐power fields (HPF), which corresponded to 1.6 mm^2^ on our microscope, and was counted jointly by two neuropathologists in the hotspot area. Meningioma subclassification was performed in accordance with the current WHO 2021 classification. For each MAM + M case, each component was macrodissected and molecularly analyzed (fluorescence in situ hybridization—FISH, DNA sequencing, and DNA‐methylation profiling).

### 2.3 Immunohistochemistry

Unstained 3‐μm‐thick slides of formalin‐fixed, paraffin‐embedded (FFPE) tissues were obtained and submitted for immunostaining with an automated stainer (Dako Omnis, Glostrup, Denmark). The following primary antibodies were used: a marker of meningioma SSTR2a (1:200, clone UMB1, Abcam, Cambridge, UK), GFAP (1:200, clone 6F2, Dako, Glostrup, Denmark), INI1 (1:50, clone 25/BAF47, BD‐Biosciences, Erembodegem, Belgium), BAP1 (1:100, clone C4, Diagomics, Blagnac, France) which may be lost in a subset of meningiomas and meningiomatosis, and Ki‐67 (1:200, clone MIB‐1, Dako, Glostrup, Denmark). External positive and negative controls were used for all antibodies and staining.

### 2.4 FISH analyses

A FISH study was performed on interphase nuclei according to the standard procedures and the manufacturer's instructions. The *SMARCB1* and *NF2* gene copy numbers were assessed using the following centromeric and locus‐specific probes: Z‐2178‐50 (Zytovision, Bremerhaven, Germany) and FG0003 (Abnova, Taipei, Taiwan). A deletion was defined if more than 30% of nuclei presented no signal for *CDKN2A* locus (at least 100 nuclei counted for each case). Results were recorded using a DM600 fluorescence imaging microscope (Leica Biosystems, Richmond, IL) fitted with appropriate filters, a charge‐coupled device camera, and digital imaging software from Leica (Cytovision, v7.4).

### 2.5 DNA sequencing

The design of a custom next‐generation sequencing (NGS) panel called DRAGON (for the Detection of Relevant Alterations in Genes involved in Oncogenetics by NGS) and marketed by Agilent under the name of SureSelect CD Curie CGP has been developed specifically for the molecular analysis of lesions. It is composed of 571 genes of interest in oncology from diagnostic, prognostic, and molecular therapy points of view, including, the full sequence of *NF2*, *SMARCB1*, *AKT1 SMO*, and *PIK3CA*, and the hotspot region of interest at the codon 409 of *KLF4*. The nucleotide sequence (variant calling is performed using Varscan2) as well as the number of copies (deletion and focal amplification) were explored. 50 ng of DNA input was extracted from FFPE lesions and used to prepare the library with the Agilent SureSelect XT‐HS preparation kit in accordance with the manufacturer's protocol. The design uses 571 genes and an additional backbone of probes across the whole genome with an average resolution of one probe every 200 Kb. This allowed us to determine a ploidy and an estimated cellularity, together with a genomic profile spanning every chromosome. The copy number profile for each case was estimated using a combination of homemade R scripts and a facets package (v0.6.0) with a sex‐specific unmatched‐germline control, previously sequenced using the same panel for normalization. Thirty‐two DNA were sequenced per 2 × 100 Sp flowcell of the NovaSeq Sequencer (Illumina) to reach an average depth of 1500X and a minimum depth of 100X on the region of interest.

### 2.6 DNA methylation profiling

DNA was extracted from FFPE tissue samples (a macrodissection of FFPE blocks was performed in cases of low cellular density) using the Qiagen DNeasy Blood & Tissue Kit (Cat NO./ID 69504), according to the manufacturer's instructions. 500 ng of DNA was extracted from each tissue sample. The DNA was sent to the Genotyping facility of the German Cancer Research Center (Heidelberg, Germany). All patient samples were analyzed using either Illumina Infinium Methylation EPIC or HumanMethylation450 BeadChip arrays according to the manufacturer's instructions. Affiliation predictions were obtained from a DNA methylation‐based classification web platform for CNS tumors (www.molecularneuropathology.org, version 11b4). Next, a t‐distributed stochastic neighbor embedding (t‐SNE) analysis was performed and compared with the genome‐wide DNA methylation profiles from the brain tumor reference cohort [4], as well as with a series of 30 pediatric meningiomas from our center. Data was generated by the DKFZ Genomics and Proteomics Core Facility (Heidelberg, Germany) as previously described [4]. Dimensionality reduction was then performed using the uniform manifold approximation and projection method (uwot R package) with the following non‐default parameters: n_neighbors = 10, spread = 2, min‐dist = 0.2.

## 3 RESULTS

### 3.1 Clinical characteristics

The main results are summarized in Table 1. All patients included in this series were pediatric, except one (#2). The median age at diagnosis was 10.0 years (patients' ages ranged from 9 to 28 years) for patients with pure MAM and 9.0 years (ranging from 1 to 12 years) for patients with MAM + M. The male/female sex ratio was 1.3 (4 males and 3 females) and 3.0 (3 males and 1 female), respectively in pure MAM and MAM + M. Lesion locations varied, with the frontal lobes being the most common location (6/11 cases, 55%). All cases, except one (case #7), were revealed by seizures. The remaining patient was followed for a medulloblastoma, non‐WNT/non‐SHH, treated by surgery, chemotherapy, and craniospinal radiation therapy, and presented during the radiological follow‐up (6 years after) an asymptomatic supratentorial MAM. There was no context of neurofibromatosis type 2 in the whole cohort. Another patient (#11) presented in the same surgical time, an atypical rhabdoid and teratoid tumor (AT/RT) component, *SMARCB1*‐deficient, a meningioma, and a MAM. All three lesions were located in the left parietal lobe. There was no known *SMARCB1* germline alteration. All patients, except one (case #3) underwent total resection. No patient received adjuvant treatment. Outcome data was available for all patients included in the cohort. Only one (9%) patient (case #3) had progression (after subtotal resection), with a mean progression‐free survival of 124.1 months. All patients were alive at the end of follow‐up (median overall survival: 193 months, ranging from 41 to 690 months).

**TABLE 1:** Summary of clinical, histopathological and molecular findings.

Case	Age	Sex	Histopathological diagnosis (grade)	Symptoms	Location	Medical history	OS (months)	DNA‐methylation profiling v12.5 (calibrated score)	CNV/FISH analyses	DNA‐sequencing analyses
1	9	M	MAM with a fibroblastic‐like pattern	Epilepsy	Right temporal	0	98.5	Ganglioglioma (0.19)	Flat	*KMT2C*
2	28	F	MAM with a fibroblastic‐like pattern	Epilepsy	Left temporal	0	371	Control hemispheric tissue (0.12)	Flat	WT^a^
3	10	M	MAM with a fibroblastic‐like pattern	Epilepsy	Left frontal	0	473.33	Glioblastoma, IDH‐wildtype (0.09)	Flat	WT^a^
4	10	M	MAM with cellular areas	Epilepsy	Parietal	0	68.75	Desmoplastic infantile ganglioglioma/astrocytoma (0.16)	Flat	WT^a^
5	9	M	MAM with cellular areas	Epilepsy	Left temporal	0	301.83	Desmoplastic infantile ganglioglioma/astrocytoma (0.14)	Hemizygous del. 22q, del. 13	WT^a^
6	9	F	MAM with cellular areas	Epilepsy	Right frontal	0	147.92	Pilocytic astrocytoma, hemispheric (0.18)	Gain 1q	WT^a^
7	15	F	MAM with cellular areas	Fortuitous	Left frontal	Medulloblastoma	41.33	Meningioma, subtype benign, subclass 3 (0.30)	Hemizygous del. 22q, del. 1q	WT^a^
8	11	F	Meningothelial meningioma (1)	Epilepsy	Left frontal	0	470.5	Meningioma, subtype benign, subclass 3 (0.09)	Hemizygous del. 22q, gain 3p	WT^a^
	11	F	MAM	Epilepsy	Left frontal	0	193	Desmoplastic infantile ganglioglioma/astrocytoma (0.21)	Hemizygous del. 22q, gain 3p	WT^a^
9	7	M	Atypical meningioma (2)	Epilepsy	Right frontal	0	690.33	Meningioma, subtype benign, subclass 1 (0.60)	Hemizygous del. 22q, del. 1q, del. 17p	WT^a^
	7	M	MAM	Epilepsy	Right frontal	0	165.92	Desmoplastic infantile ganglioglioma/astrocytoma (0.13)	Hemizygous del. 22q, del. 1q, del. 17p	WT^a^
10	12	M	Atypical meningioma (2)	Epilepsy	Convexity	0	98.5	Meningioma, subtype benign, subclass 3 (0.30)	Hemizygous del. 22q, *EGFR* amplification, gain 5p	WT^a^
	12	M	MAM	Epilepsy	Convexity	0	371	Supratentorial ependymoma, ZFTA fusion‐positive (0.08)	Hemizygous del. 22q	WT^a^
11	1	M	Atypical meningioma (2)	Epilepsy	Left parietal	AT/RT	473.33	Meningioma, subtype benign, subclass 3 (0.99)	Hemizygous del. 22q, del. 1p, del. 2p	WT^a^
	1	M	MAM	Epilepsy	Left parietal	AT/RT	68.75	Teratoma (0.33)	Hemizygous del. 22q, del. 2p	WT^a^

Abbreviations: AT/RT, atypical teratoid and rhabdoid tumor; CNV, copy number variations; del., deletion; F, female; M, male; MAM, meningioangiomatosis; OS, overall survival; WT, wildtype.

^a^ Wildtype for all the 571 genes of the panel used.

### 3.2 Radiological characteristics

**FIGURE 1:**
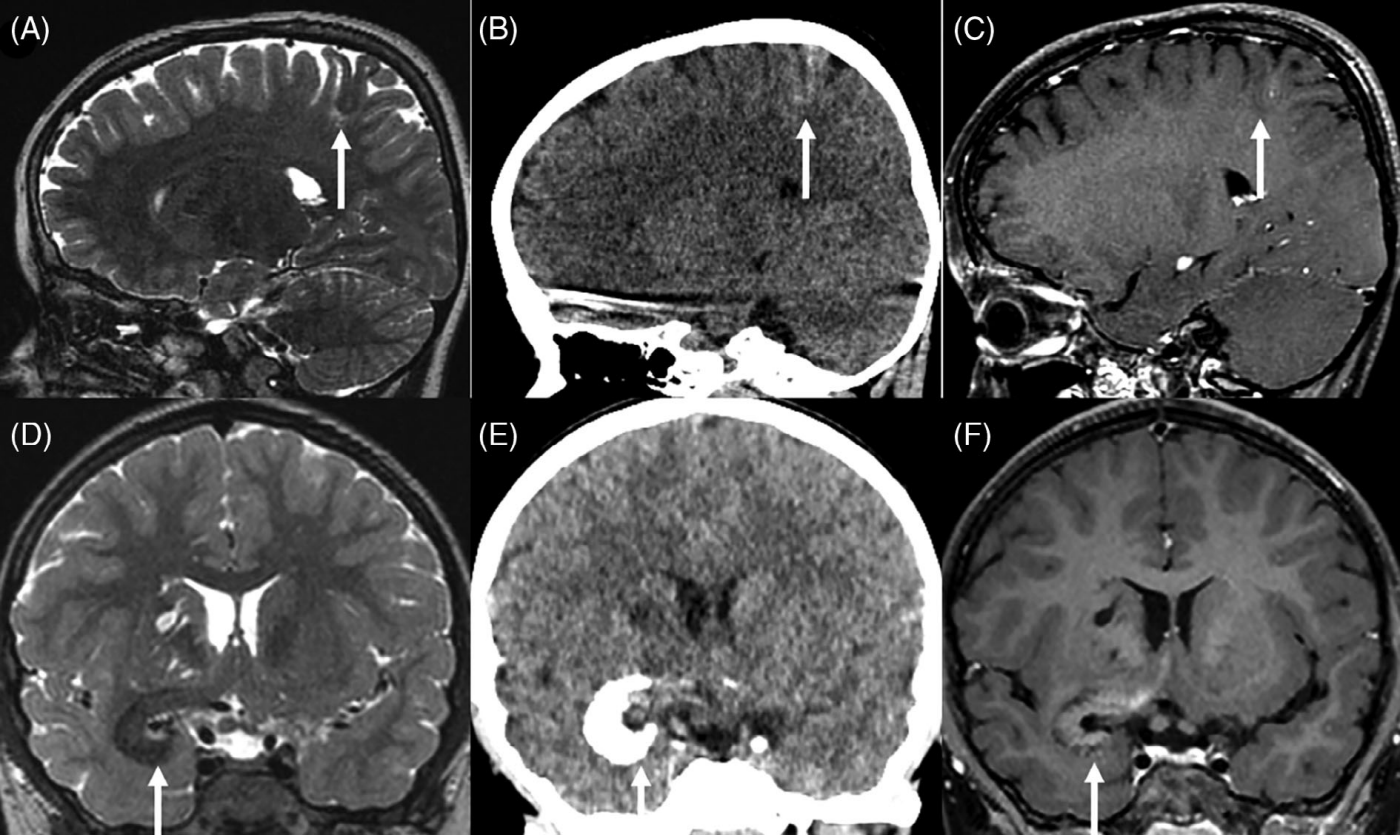
Neuroradiological features of meningioangiomatoses. Magnetic resonance imaging of patient #4, showing a cortical T2‐weighted low intensity signal with a subcortical high intensity signal (A), high density on computed tomography with microcalcification (B) and very slight contrast enhancement (C). Magnetic resonance imaging of patient #1, depicts a thick cortex with T2‐weighted low intensity signal and enlarged Virchow‐Robin spaces in the basal ganglia (D), coarse calcification on computed tomography (E) and contrast enhancement (F).

Preoperative imaging was available for 10/11 cases (see Supplementary Table 1 and Figure 1). All lesions affected the cortex and 8/10 cases (80%) were associated with subcortical white matter abnormalities. MRI showed thickening of the cortex, with a T2‐weighted low signal in 7/10 cases (70%) and an intermediate signal in 1/10 cases. The T1‐weighted signals were slightly higher than the normal cortex in 6/10 cases, and intermediate in 3/10 cases. An existence of cortical calcifications in all the cases with available CT (7/7) may explain this phenomenon. Only one case (#7) exhibited a high cortical signal on T2‐weighted images and low signal on T1‐weighted images. This case was detected during postoperative medulloblastoma follow‐up, without any symptoms. The unique imaging characteristics may be attributed to the early detection of the radiological diagnosis before MAM calcification. Contrast enhancement varied from none to high (similar to choroid plexus). When available, diffusion was not restricted (high or intermediate apparent diffusion coefficient, 8/10 cases). Cerebral blood flow was low on arterial spin labeling perfusion MRI (7/10 cases).

In 80% of cases, white matter involvement manifested as a subcortical T2‐weighted high signal; however, in case #11, the T2‐weighted low signal and calcifications extended into the white matter toward the lateral ventricles. Three cases presented with enlarged Virchow‐Robin spaces. These spaces varied in size and location, with some being subcortical with minor extensions (#11), while others were very large extending from the frontal cortex to the lateral ventricle (#3) or found within the basal ganglia, associated with an infiltration around the proximal middle cerebral artery (#1).

**FIGURE 2:**
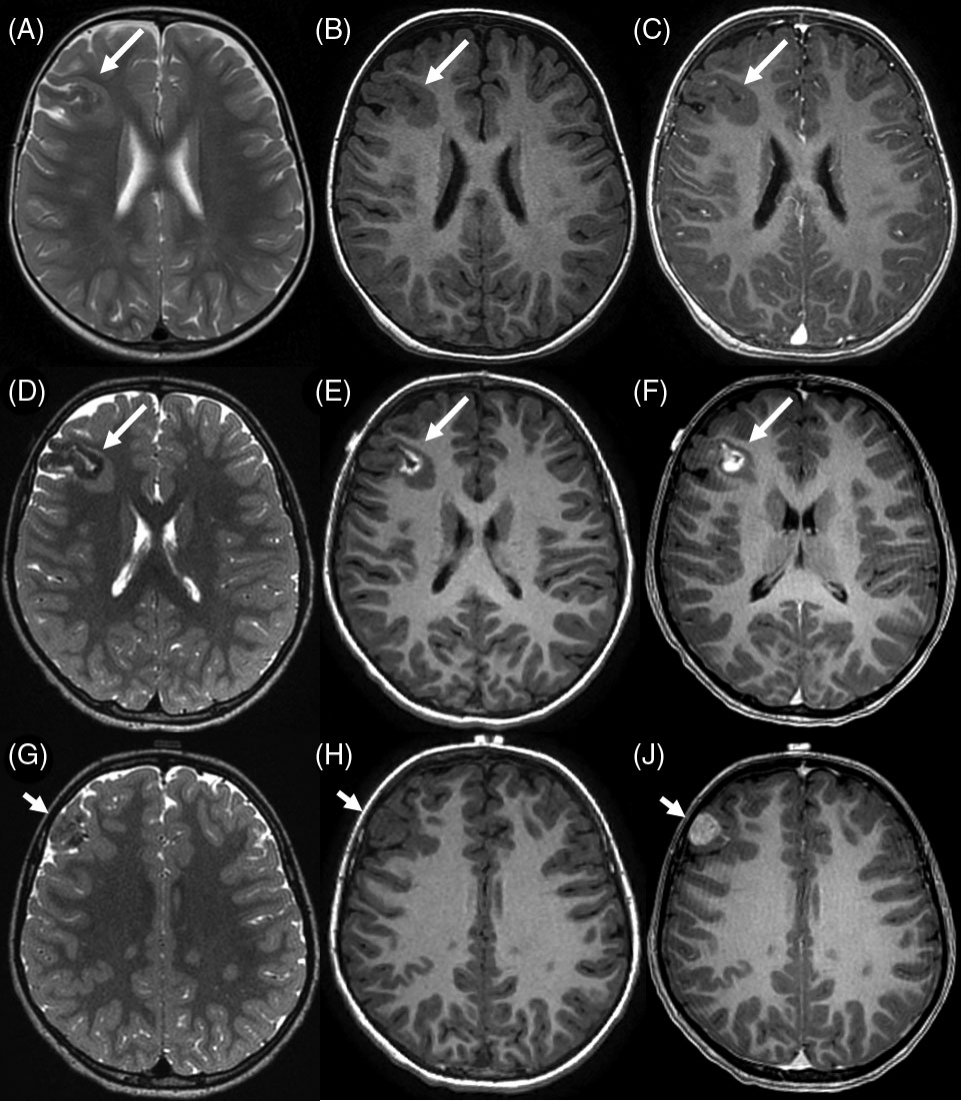
Neuroradiological features of Case #9. Magnetic Resonance Imaging performed at diagnosis at the age of 2 (A)–(C) and before surgery at the age of 7 (D), (I). The right frontal meningioangiomatosis is observed as cortical thickening with T2‐weighted low intensity signal (A), (D), an intermediate signal on T1‐weighted images at diagnosis (B), but high intensity signal during follow‐up (D), consistent with calcification on computed tomography (not shown), and very small contrast enhancement (F). Additionally, a small subcortical T2‐weighted high intensity signal is present (D). An extra‐axial mass with T2 and T1‐weighted low intensity signal (G), (H) and strong contrast enhancement (I) is indicative of an associated meningioma. It was not visible on the initial Magnetic Resonance Imaging scan (not shown).

MRI was available for 3/4 MAM + M cases. The cortical MAM presented similarities to others but was connected to an extra‐axial nodular mass in the immediate vicinity, without dural thickening. These masses showed imaging features typical of meningioma, with strong contrast enhancement. In one instance (#9, Figure 2), the meningioma was not detected during the radiological diagnosis, it appeared after a follow‐up of 3 years with subsequent growth after 2 years, and a gradual radiological progression of the MAM.

### 3.3 Histopathological and Immunohistochemical characterization

**FIGURE 3:**
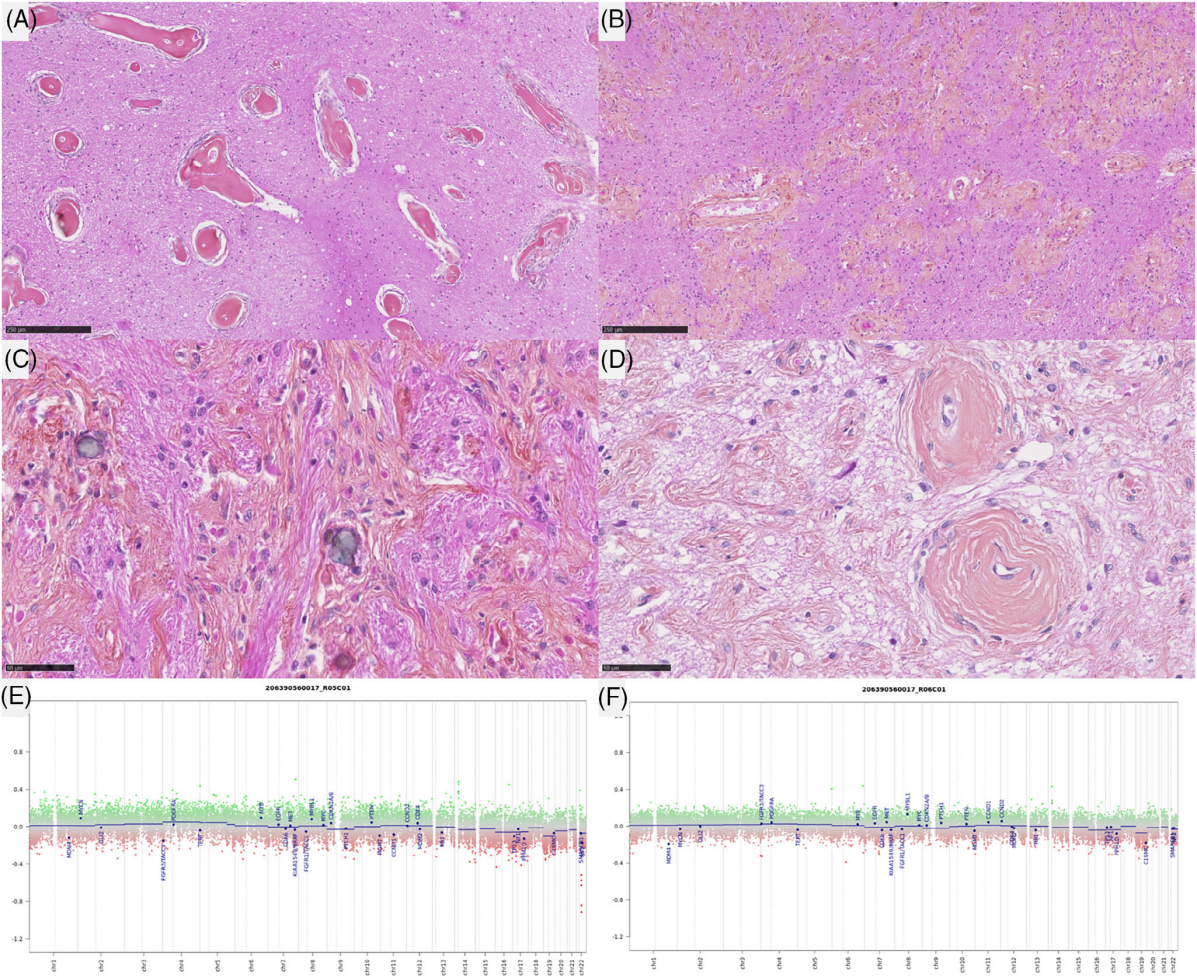
Histopathological and molecular features of Case #1. (A)–(D) Pure meningioangiomatosis with a fibroblastic‐like pattern characterized by fibrous spread along the Virchow‐Robin spaces with few cells (HPS, magnification x80 for (A), (B), and ×400 for (C), (D). (E), (F) Flat copy number variations with only a focal deletion on 22q. Black scale bars represent 250 μm (A), (B) and 50 μm (C), (D). HPS, hematoxylin phloxin saffron.

**FIGURE 4:**
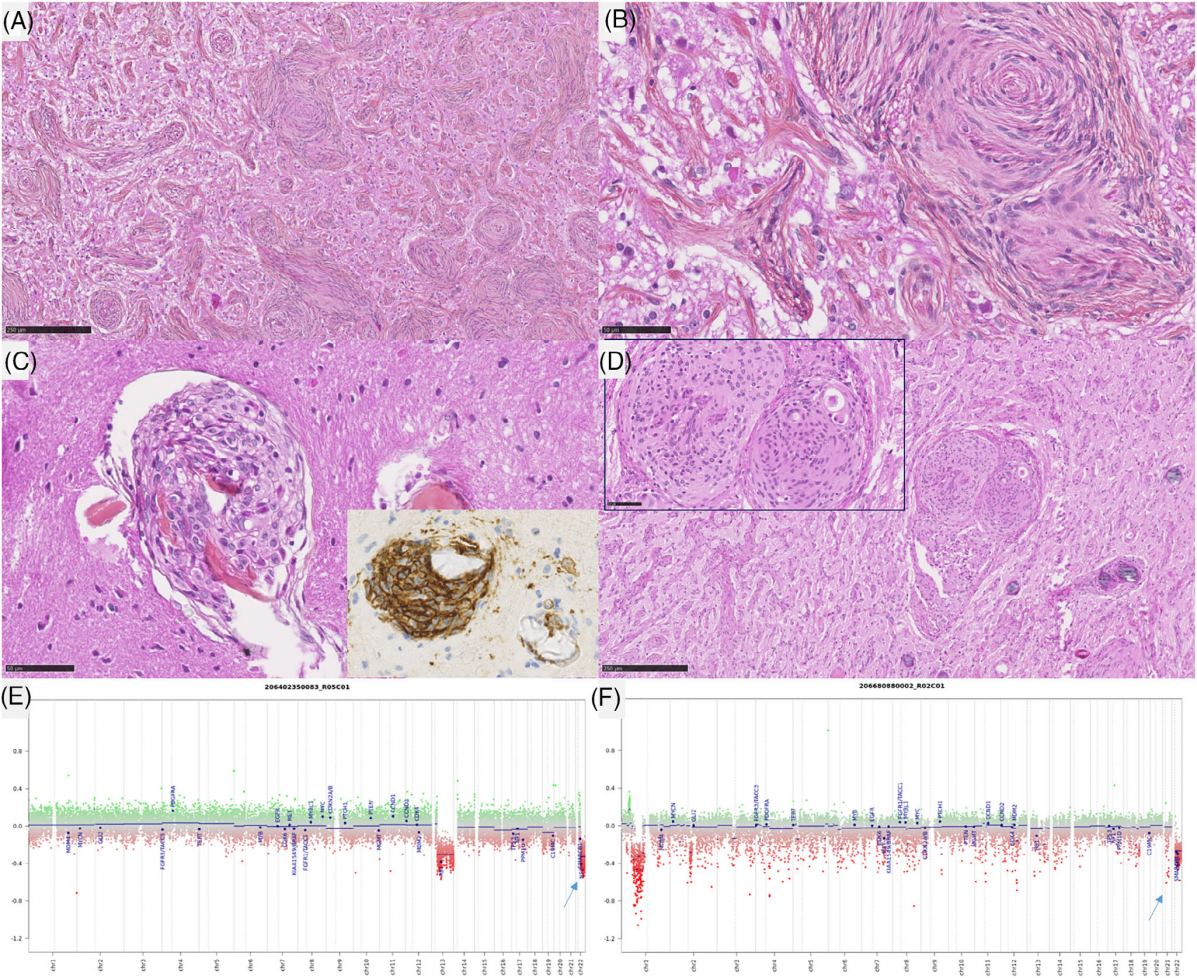
Histopathological and molecular features of Case #6. (A)–(D) Pure meningioangiomatosis with a cellular pattern characterized by fibrous spread along the Virchow‐Robin spaces with cells and meningothelial nodules (HPS, magnification ×80 for (A)–(D), and ×400 for (b), (C), and insert), stained by SSTR2a (insert ×400). (E), (F) Copy number variations showing a hemizygous deletion of chromosome 22q. Black scale bars represent 250 μm (A)–(D) and 50 μm (B), (C). HPS, hematoxylin phloxin saffron.

In the MAM cohort, two morphological patterns were observed. First, three lesions (cases #1–3) presented low cellular density, composed of a fibrous infiltration of the Virchow‐Robin spaces (MAM with a fibroblastic‐like pattern) (Figure 3A–D). The four other cases (cases #4–7) presented an association of fibrous patterns and small nodules of meningothelial cells (MAM with cellular areas) (Figure 4A–D). There were whorls and psammoma bodies, and the meningothelial cells did not present cytological atypia or mitoses. In these cases, we observed a transition between a fibrous component at the surface of the leptomeninges with a fibrous spread in the Virchow‐Robin spaces and the formation of meningothelial nodules (Supplementary Figure 1). Necrosis was absent in all MAM cases. Using IHC, INI1, and BAP1 immunoexpressions were retained in all MAM cases. The MIB1 labeling index was weak (less than 1% in all cases) and all lesions expressed SSTR2a.

In the MAM + M cohort, all cases presented a morphological spectrum from a paucicellar fibrous infiltration of the Virchow‐Robin spaces to the formation of a circumscribed meningothelial proliferation, corresponding to a meningioma. All meningiomas were of a meningothelial subtype. Three of them presented 4, 5, and 6 mitoses per 1.6 mm^2^ (grade 2), whereas the remaining case was grade 1. There was no brain parenchyma invasion (no irregular infiltration or protrusion of tumor cells into the underlying GFAP‐positive parenchyma). Using IHC, INI1, and BAP1 immunoexpressions were retained in all MAM + M cases. The MIB1 labeling index was weak in the MAM component (less than 1% in all cases) whereas it varied from 1% to 8% in the meningioma component. In all lesions, both components (MAM and meningiomas) expressed SSTR2a.

### 3.4 Molecular results

DNA‐sequencing analyses failed to reveal any mutation *NF2*, *AKT1*, *KLF4*, *SMO*, or *PIK3CA* genes in the whole cohort (pure MAM, and in both components of MAM + M). A hemizygous deletion of chromosome 22q was detected in 2/7 pure MAM (cases #5 and 7) and in all MAM + M (presence of the deletion in both components) (Figure 4E, F). An inactivating nonsense *KMT2C* mutation with an allele frequency of 4.38% was evidenced in one pure MAM (case #1). Concerning the case #11, the hemizygous deletion of chromosome 22q included *SMARCB1* gene, and no alteration, no nucleotidic mutation or structural variant such as deletion, of *SMARCB1* gene, was detected in the MAM and the meningioma (whereas the AT/RT component harbored a homozygous deletion of *SMARCB1* gene). FISH analyses for *NF2* and *SMARCB1* failed to reveal any deletion in MAM cases which did not present a deletion of chromosome 22q on CNV (Figure 3E, F). MAM with a cellular pattern presented additional CNV alterations such as losses of chromosomal material on chromosome 1q (case #7) and chromosome 13q (case #5). MAM associated to meningiomas presented additional CNV alterations such as losses of chromosomal material on chromosome 1q (case #9) and chromosome 2 (case #11), or gain on chromosome 3p (case #8) and chromosome 5 (case #10). These alterations were also found in the meningioma component except for the gain of chromosome 5 of the case #10 but, interestingly, a focal amplification of *EGFR* appeared in the meningioma component in this case (confirmed by DNA‐methylation profiling). The size of the loss of chromosome 22q differ from one case to another. For one case (case #7), the entire chromosome 22 was lost, for some cases, the chromosome 22q loss start from chromosome band 22q11.22 (case #9) or 22q11.23 (case #10) to the end of the chromosome 22q (hence encompassing both *SMARCB1* and *NF2*), and finally for one interesting case (case #8), the deletion started from chromosome band 22q12.1 to the end of chromosome 22q hence encompassing *NF2* but not *SMARCB1*. This last result hence defined a minimal deleted region that include *NF2* gene that seems the most important for MAM development but not *SMARCB1*.

**FIGURE 5:**
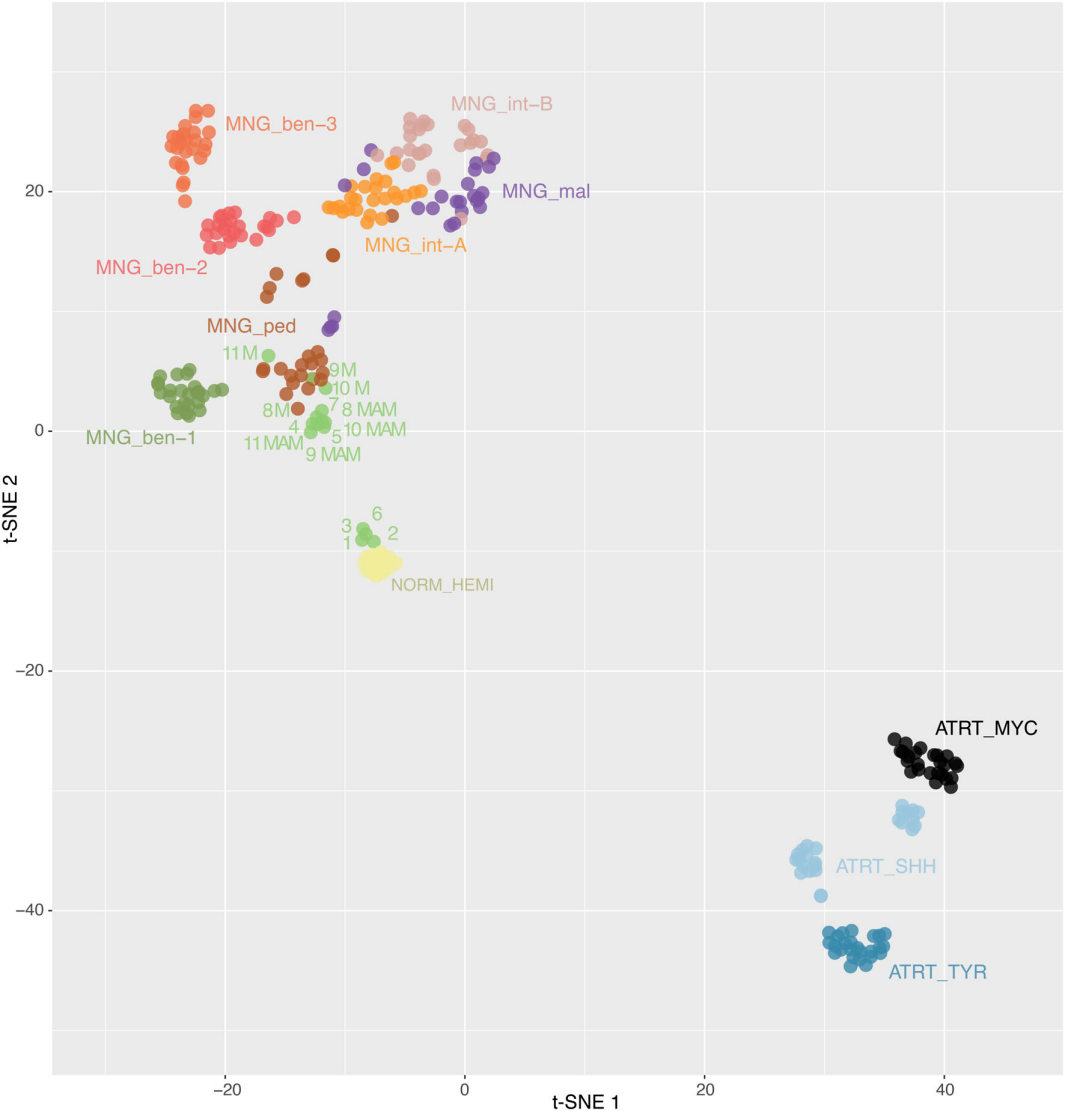
DNA methylation‐based t‐distributed stochastic neighbor embedding distribution. Our 7 pure MAM (#1–7) and MAM associated to meningiomas (#8–11) were compared to reference samples from the Heidelberg cohort belonging to the atypical teratoid and rhabdoid tumor (ATRT) (ATRT_MYC), ATRT_SHH, ATRT_SHH, Adult meningiomas, subtype benign‐1 (MNG_ben‐1), adult meningiomas, subtype benign‐2 (MNG_ben‐2), adult meningiomas, subtype benign‐3 (MNG_ben‐3), adult meningiomas, subtype intermediate A (MNG_int‐A), adult meningiomas, subtype intermediate B (MNG_int‐B), adult meningiomas, subtype malignant (MNG_mal), pediatric meningiomas (pedMNG), control tissue, cerebral hemisphere (NORM_HEMI).

According to the DNA methylation‐based classification and the DKFZ Classifier (version 12.5), none of the lesions were classifiable (calibrated scores for DNA methylation class <0.9), except one meningioma (case #11, which classified as a benign meningioma, with a calibrated score of 0.99). A t‐SNE analysis was performed to compare the genome‐wide DNA methylation profiles of pure MAM, MAM + M, pediatric meningiomas, and the different methylation classes (MC) of adult meningiomas. Most cases (three pure MAM and the four pairs of MAM + M) clustered within close proximity to the pediatric meningioma methylation class (Figure 5 and Supplementary Figure 2). Interestingly, meningiomas associated with MAM clustered within the MC of pediatric meningiomas. Moreover, MAM (pure or associated with meningiomas) formed a distinct cluster from other meningioma MC. The four remaining cases (#1, 2, 3, and 6) clustered in close vicinity with control hemispheric tissue.

## 4 DISCUSSION

**FIGURE 6:**
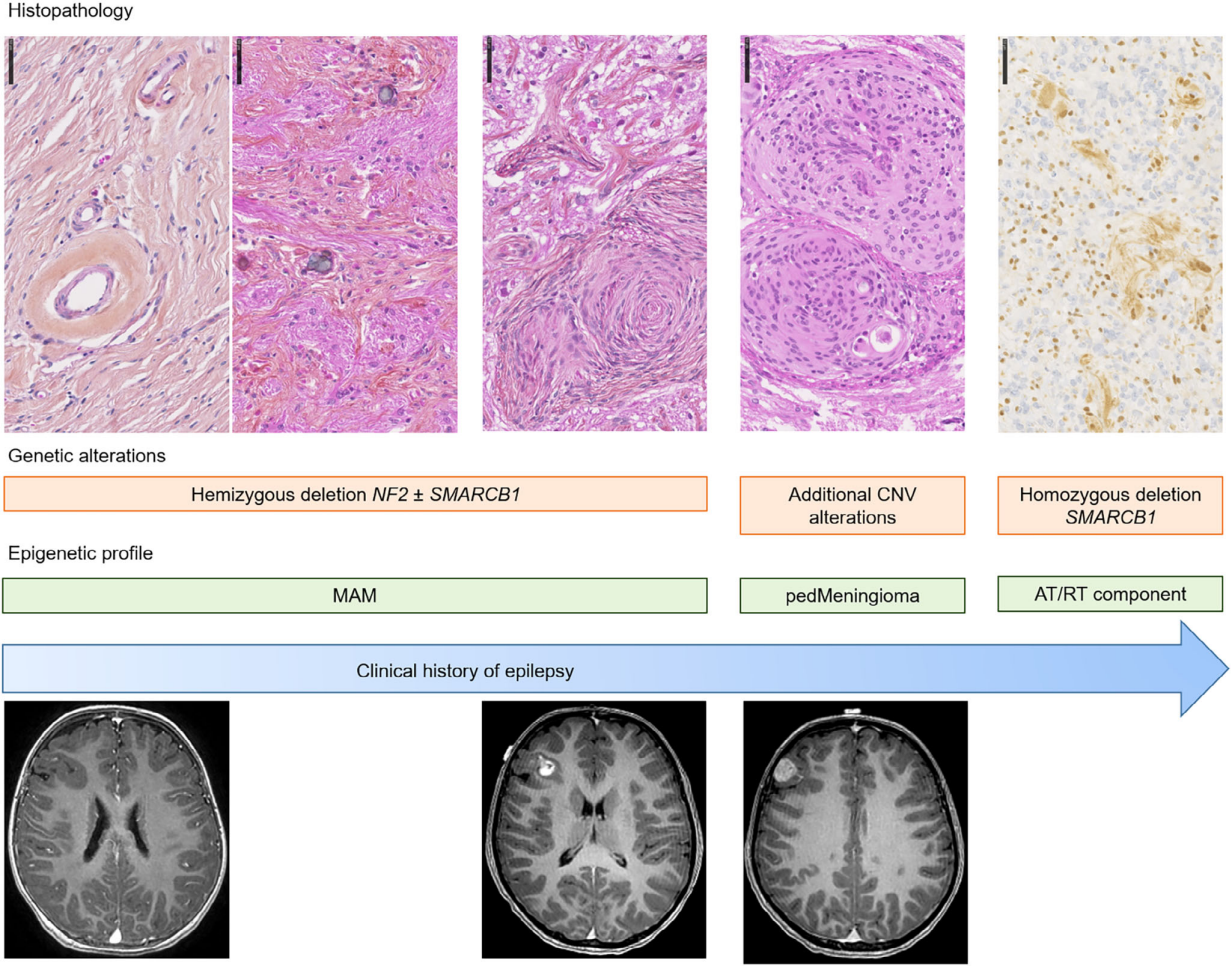
Summary of meningioangiomatosis and tumor spectrum. AT/RT, atypical teratoid and rhabdoid tumor; CNV, copy number variations; MAM, meningioangiomatosis; ped, pediatric.

The first description of MAM was in 1915 and was initially described as a lesion encountered in the context of neurofibromatosis type 2 (see review in [3]) [5, 6]. Since then, sporadic cases have been reported, and MAM associated with tumors (mainly meningiomas) have been reported in the literature [1, 7, 8, 9, 10]. The neoplastic origin of MAM remains debated and the WHO classification does not include it in the meningioma chapter. The recent literature analysis and the current study bring several arguments in favor of a neoplastic origin for MAM. MAM are mainly isolated (without a meningioma), grow slowly in the brain parenchyma, and are responsible for epilepsy, which becomes refractory. Moreover, rare observations of progressions (but only after biopsy or partial resection) (cf., review in [4]) and multifocal forms have been reported [1, 11]. The current study evidenced that a subset of pure MAM (2/7, 29%), and all MAM + M harbored a hemizygous deletion of chromosome 22q (including the *NF2* gene), which is the most frequent alteration in meningiomas (adult and pediatric) [2, 12, 13, 14, 15]. Interestingly, this deletion was present in both components (MAM and meningioma) supporting the argument of a neoplastic origin for MAM. The current series showed that MAM cases did not present any mutation described for the *NF2*, *AKT1*, *KLF4*, *SMO*, or *PIK3CA* genes. The fact that these alterations have been reported in adults and are absent in pediatric meningiomas, may explain why they are not found in MAM, which mainly affect children [13]. Another potential argument for a neoplastic origin for MAM may be that one case of the current series harbored a pathogenic variant of the *KMT2C* gene, which was previously reported in a subset of chordoid meningiomas [11]. Based on epigenetic profiling, we showed that the main subgroup of lesions (three pure MAM and the four pairs of MAM + M) did, in fact, cluster together, and were in close vicinity to the pediatric meningioma MC. Because DNA methylation profiles are thought to represent a combination of both somatically acquired DNA methylation changes and a signature reflecting the cell of origin [16], it seems that MAM belong to the lesional spectrum of pediatric meningiomas, potentially as a neoplastic lesion. The absence of an alteration in a subset of pure MAM (3/7 cases) and the close vicinity of the DNA‐methylation profile with control hemispheric tissue may be related to the low cellular density of these lesions that include normal brain parenchyma. Moreover, the current series showed that, even though some molecular alterations are present in both MAM and M components, the meningioma may present additional alterations. We can hypothesize that if the MAM component was a brain invasion or Virchow‐Robin space involvement of a meningioma, there would be additional molecular alterations and histopathological features of aggressiveness in this component, which was not the case. In the cellular pattern of MAM and in the MAM component of mixed forms, 3/7 cases presented additional CNV alterations (losses of chromosomal material on chromosomes 1 and 5), that are also found in pediatric meningiomas [13] and in the meningioma component of mixed cases. Considering these results, we can hypothesize that there exists a histomolecular continuum between the pure MAM (primarily represented as a hypointense lesion on T1 weighted sequences with hyper signal in T2 weighted sequences, composed of a fibrous spread in the Virchow‐Robin spaces, showing calcifications on MRI and becoming more cellular with the constitution of cellular nodules harboring a 22q hemizygous deletion) and the MAM associated with authentic meningiomas (Figure 6). Because those lesions are cortical, symptoms include epileptic seizures. Consequently, we can suggest that histopathological findings encountered in surgical samples may depend on the age of patients and the age of epilepsy onset, with the constitution of meningiomas over the long run, being associated with pharmaco‐resistant seizures.

Previously, MAM have been described as being associated with AT/RT or tumors having CNS *SMARCB1‐*alterations [17, 18]. However, molecular data (including 22q status in the two components, germline status of *SMARCB1*, and epigenetic analyses) are lacking for these cases. The current study showed that MAM and meningioma components harbored only a hemizygous deletion of chromosome 22q including the *SMARCB1* gene, whereas a homozygous deletion was present in the AT/RT component. The DNA‐methylation profiling of these three components are distinct. This may illustrate the fact that an additional genetic event may be responsible for a subset of patients with MAM where AT/RT later appears. Moreover, meningiomas belong to the tumor spectrum for cases of rhabdoid tumor predisposition syndrome [19, 20]. This hypothesis is reinforced by the fact that this AT/RT component was classified as an AT/RT subtype MYC, which is known to be similar to extracranial forms of rhabdoid tumors (non‐neuronal), and in this case, potentially shares the same cellular origin as MAM and meningiomas.

To conclude, the current work brought additional findings arguing a potential neoplastic nature for MAM, which might be isolated or progress to form a meningioma. If further series confirm these data, MAM will need to be included in the meningioma chapter of a future version of the WHO Classification of CNS Tumors.

## AUTHOR CONTRIBUTIONS

ATE, VDR, NB, OA, AR, JPTB, KB, FB, and SP compiled the MRI and clinical records; ATE, AM, FC, and PV conducted the neuropathological examinations; JMP, PS, and FS conducted the molecular studies; ATE, JMP, PS, LH, and PV drafted the manuscript; all authors reviewed the manuscript.

## CONFLICT OF INTEREST STATEMENT

The authors declare that they have no conflict of interest directly related to the topic of this article.

## ETHICS STATEMENT

This study was approved by the GHU Paris Psychiatry Neurosciences, Sainte‐Anne Hospital's local ethics committee. All the patients' parents or legal guardians signed informed consent forms before treatment was started. We obtained human subjects approval from our institutional review board.

## Supporting information


**Supplementary Figure 1** Transition between a fibrous component at the surface of the leptomeninges with a fibrous spread in the Virchow‐Robin spaces and the formation of meningothelial nodules (HPS, magnification ×20 for a, and ×400 for b, ×80 for c, and ×200 for D). Black scale bars represent 1 mm (a), 50 μm (b), 250 μm (c) and 100 μm (d). HPS: Hematoxylin Phloxin Saffron.

**Supplementary Figure 2** Dimensionality reduction with uniform manifold approximation and projection (UMAP). Our 7 pure MAM (#1–7) and MAM associated to meningiomas (#8–11) were compared to reference samples from the Heidelberg cohort belonging to the atypical teratoid and rhabdoid tumor (ATRT) (ATRT_MYC), ATRT_SHH, ATRT_SHH, Adult meningiomas, subtype benign‐1 (MNG_ben‐1), Adult meningiomas, subtype benign‐2 (MNG_ben‐2), Adult meningiomas, subtype benign‐3 (MNG_ben‐3), Adult meningiomas, subtype intermediate A (MNG_int‐A), Adult meningiomas, subtype intermediate B (MNG_int‐B), Adult meningiomas, subtype malignant (MNG_mal), Pediatric meningiomas (pedMNG), Control tissue, cerebral hemisphere (NORM_HEMI).

**Supplementary Table 1.** Detailed radiological characteristics of included patients.

**Data S1:** Supporting Information

## Data Availability

Data sharing is not applicable to this article as no new data were created or analyzed in this study.

## References

[1] Cui H , Shi H , Chen X , Wang W , Lai R , Han A . Clinicopathological features of meningioangiomatosis associated with meningioma: a case report with literature review. Case Rep Oncol Med. 2012;2012:296286. 10.1155/2012/296286 23198201 PMC3502778

[2] Dono A , Pothiawala AZ , Lewis CT , Bhattacharjee MB , Ballester LY , Tandon N . Molecular alterations in Meningioangiomatosis causing epilepsy. J Neuropathol Exp Neurol. 2021;80:1043–1051. 10.1093/jnen/nlab095 34580720 PMC8921655

[3] Roux A , Zanello M , Mancusi RL , Still MEH , Nascimento FA , Tauziede‐Espariat A , et al. Meningioangiomatosis: multimodal analysis and insights from a systematic review. Neurology. 2021;96:274–286. 10.1212/WNL.0000000000011372 33361266

[4] Capper D , Jones DTW , Sill M , Hovestadt V , Schrimpf D , Sturm D , et al. DNA methylation‐based classification of central nervous system tumours. Nature. 2018;555:469–474. 10.1038/nature26000 29539639 PMC6093218

[5] Fedi M , Kalnins RM , Shuey N , Fitt GJ , Newton M , Mitchell LA . Cystic meningioangiomatosis in neurofibromatosis type 2: an MRI‐pathological study. Br J Radiol. 2009;82:e129–e132. 10.1259/bjr/56536580 19541939

[6] Omeis I , Hillard VH , Braun A , Benzil DL , Murali R , Harter DH . Meningioangiomatosis associated with neurofibromatosis: report of 2 cases in a single family and review of the literature. Surg Neurol. 2006;65:595–603. 10.1016/j.surneu.2005.09.034 16720184

[7] Deb P , Gupta A , Sharma MC , Gaikwad S , Singh VP , Sarkar C . Meningioangiomatosis with meningioma: an uncommon association of a rare entity—report of a case and review of the literature. Childs Nerv Syst. 2006;22:78–83. 10.1007/s00381-004-1074-4 16389566

[8] López JI , Ereño C , Oleaga L , Areitio E . Meningioangiomatosis and oligodendroglioma in a 15‐year‐old boy. Arch Pathol Lab Med. 1996;120:587–590. 8651864

[9] Mut M , Söylemezoğlu F , Firat MM , Palaoğlu S . Intraparenchymal meningioma originating from underlying meningioangiomatosis. Case report and review of the literature. J Neurosurg. 2000;92:706–710. 10.3171/jns.2000.92.4.0706 10761664

[10] Shi H , Zhao S , Tian X , Li Z , Huang Q , Li B . Meningioangiomatosis‐associated meningioma misdiagnosed as glioma by radiologic and intraoperative histological examinations. Brain Tumor Pathol. 2011;28:347–352. 10.1007/s10014-011-0045-1 21681536

[11] Daoud EV , Zhu K , Mickey B , Mohamed H , Wen M , Delorenzo M , et al. Epigenetic and genomic profiling of chordoid meningioma: implications for clinical management. Acta Neuropathol Commun. 2022;10:56. 10.1186/s40478-022-01362-3 35440040 PMC9020042

[12] Kim NR , Cho SJ , Suh Y‐L . Allelic loss on chromosomes 1p32, 9p21, 13q14, 16q22, 17p, and 22q12 in meningiomas associated with meningioangiomatosis and pure meningioangiomatosis. J Neurooncol. 2009;94:425–430. 10.1007/s11060-009-9879-3 19347254

[13] Kirches E , Sahm F , Korshunov A , Bluecher C , Waldt N , Kropf S , et al. Molecular profiling of pediatric meningiomas shows tumor characteristics distinct from adult meningiomas. Acta Neuropathol. 2021;142:873–886. 10.1007/s00401-021-02351-x 34495383 PMC8500891

[14] Perry A , Kurtkaya‐Yapicier O , Scheithauer BW , Robinson S , Prayson RA , Kleinschmidt‐DeMasters BK , et al. Insights into meningioangiomatosis with and without meningioma: a clinicopathologic and genetic series of 24 cases with review of the literature. Brain Pathol. 2005;15:55–65. 10.1111/j.1750-3639.2005.tb00100.x 15779237 PMC8095908

[15] Toland A , McNulty SN , Pekmezci M , Evenson M , Huntoon K , Pierson CR , et al. Pediatric meningioma: a clinicopathologic and molecular study with potential grading implications. Brain Pathol. 2020;30:1134–1143. 10.1111/bpa.12884 32716568 PMC8018050

[16] Hovestadt V , Jones DTW , Picelli S , Wang W , Kool M , Northcott PA , et al. Decoding the regulatory landscape of medulloblastoma using DNA methylation sequencing. Nature. 2014;510:537–541. 10.1038/nature13268 24847876

[17] Iorgulescu JB , Ferris S , Agarwal A , Casavilca Zambrano S , Hill DA , Schmidt R , et al. Non‐meningothelial meningeal tumours with meningioangiomatosis‐like pattern of spread. Neuropathol Appl Neurobiol. 2018;44:743–746. 10.1111/nan.12481 29495087 PMC6119531

[18] Rossi S , Brenca M , Zanatta L , Trincia E , Guerriero A , Pizzato C , et al. A pediatric intra‐axial malignant SMARCB1‐deficient desmoplastic tumor arising in meningioangiomatosis. J Neuropathol Exp Neurol. 2018;77:883–889. 10.1093/jnen/nly075 30169623

[19] Gossai N , Biegel JA , Messiaen L , Berry SA , Moertel CL . Report of a patient with a constitutional missense mutation in SMARCB1, coffin‐Siris phenotype, and schwannomatosis. Am J Med Genet A. 2015;167A:3186–3191. 10.1002/ajmg.a.37356 26364901

[20] Kehrer‐Sawatzki H , Kordes U , Seiffert S , Summerer A , Hagel C , Schüller U , et al. Co‐occurrence of schwannomatosis and rhabdoid tumor predisposition syndrome 1. Mol Genet Genomic Med. 2018;6:627–637. 10.1002/mgg3.412 29779243 PMC6081224

