# Supplementary figures and images for: A comprehensive histomolecular characterization of meningioangiomatosis: Further evidence for a precursor neoplastic lesion
Source: Brain Pathol. 2024 Apr 2;34(6):e13259. doi: 10.1111/bpa.13259 (PMC11483523; doi:10.1111/bpa.13259)

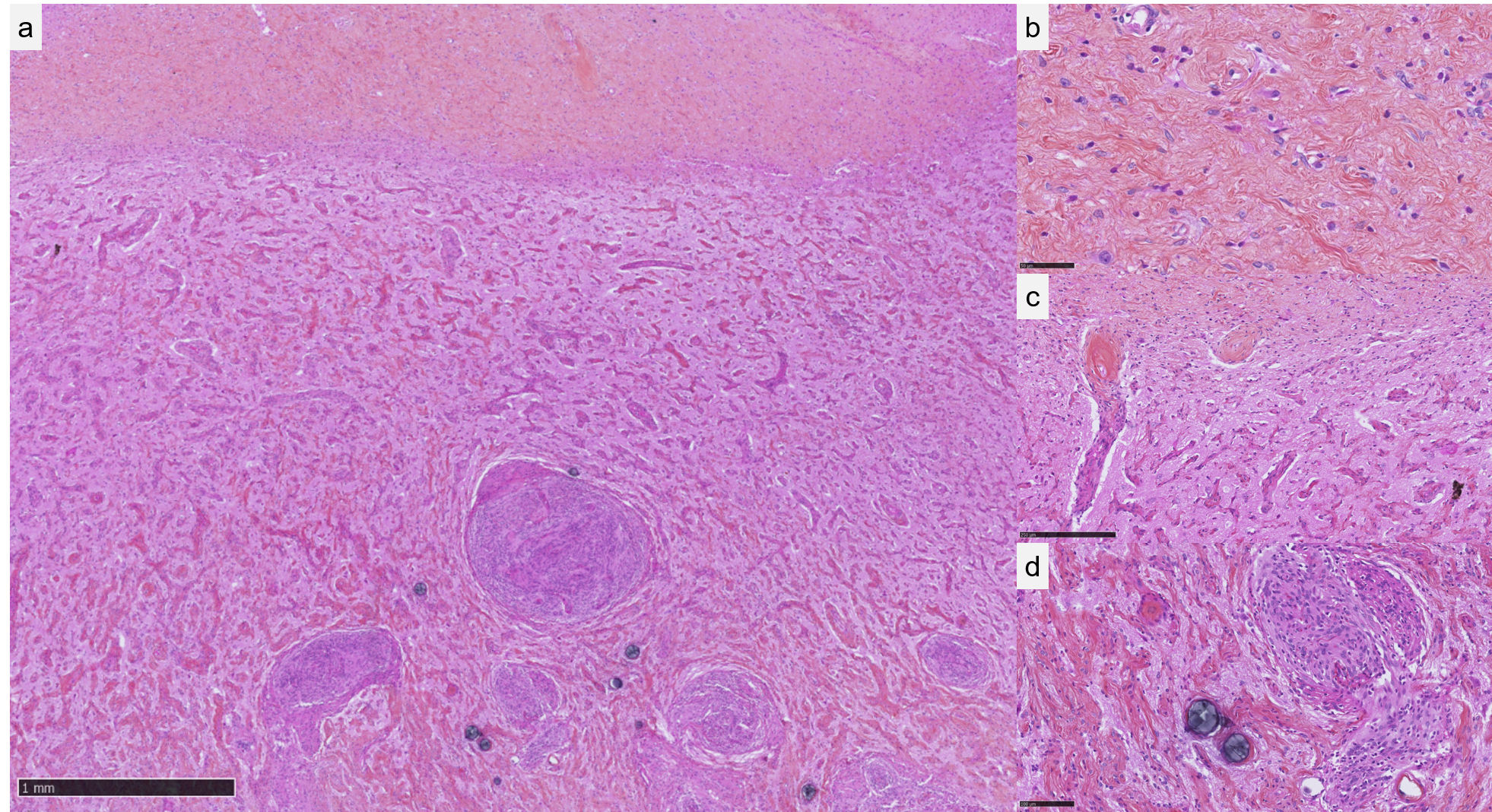

Supplement: Supplementary file 1 — Supplementary Figure 1 Transition between a fibrous component at the surface of the leptomeninges with a fibrous spread in the Virchow‐Robin spaces and the formation of meningothelial nodules (HPS, magnification ×20 for a, and ×400 for b, ×80 for c, and ×200 for D). Black scale bars represent 1 mm (a), 50 μm (b), 250 μm (c) and 100 μm (d). HPS: Hematoxylin Phloxin Saffron. [file BPA-34-e13259-s001.tif]

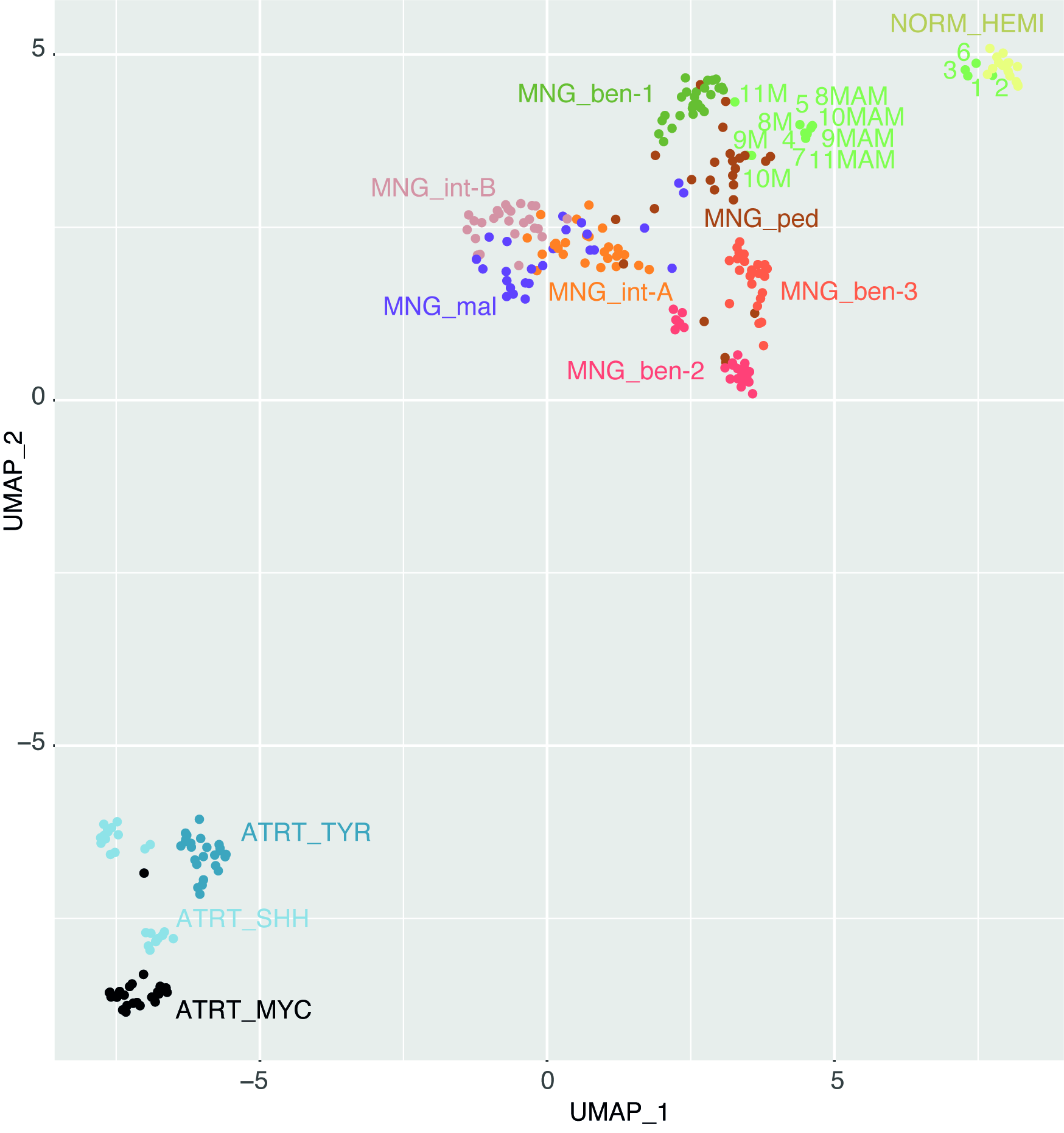

Supplement: Supplementary file 2 — Supplementary Figure 2 Dimensionality reduction with uniform manifold approximation and projection (UMAP). Our 7 pure MAM (#1–7) and MAM associated to meningiomas (#8–11) were compared to reference samples from the Heidelberg cohort belonging to the atypical teratoid and rhabdoid tumor (ATRT) (ATRT_MYC), ATRT_SHH, ATRT_SHH, Adult meningiomas, subtype benign‐1 (MNG_ben‐1), Adult meningiomas, subtype benign‐2 (MNG_ben‐2), Adult meningiomas, subtype benign‐3 (MNG_ben‐3), Adult meningiomas, subtype intermediate A (MNG_int‐A), Adult meningiomas, subtype intermediate B (MNG_int‐B), Adult meningiomas, subtype malignant (MNG_mal), Pediatric meningiomas (pedMNG), Control tissue, cerebral hemisphere (NORM_HEMI). [file BPA-34-e13259-s002.tif]
