# Supplementary material for: A comprehensive histomolecular characterization of meningioangiomatosis: Further evidence for a precursor neoplastic lesion
Source: Brain Pathol. 2024 Apr 2;34(6):e13259. doi: 10.1111/bpa.13259 (PMC11483523; doi:10.1111/bpa.13259)
Supplement: Supplementary file 3 — Supplementary Table 1. Detailed radiological characteristics of included patients. [file BPA-34-e13259-s003.docx]

| case | Lobe | Involved structures | Calcifications | T1w signal | T2w  signal | Contrast enhancement | Cysts | Meningioma |
| --- | --- | --- | --- | --- | --- | --- | --- | --- |
| 1 | Temporal | Cortex + WM | Present | Iso | Low | Moderate | Dilated VR spaces | No |
| 2 | Temporal | Cortex | NA | High | NA | NA | No | No |
| 3 | Frontal | Cortex + WM | NA | Iso | Low | None | Dilated VR spaces | No |
| 4 | Parietal | Cortex + WM | Present | High | Low | Faint | No | No |
| 5 | Temporal | Cortex + WM | Present | High | Low | Faint | No | No |
| 6 | Frontal | Cortex + WM | Present | High | Low | Strong | No | No |
| 7 | Frontal | Cortex | NA | Low | High | None | No | No |
| 8 | Frontal | Cortex + WM | Present | Iso | Low | High | No | Yes |
| 9 | Frontal | Cortex + WM | Present | High | Low | Faint | No | Yes |
| 10 | Frontal | NA | NA | NA | NA | NA | NA | NA |
| 11 | Parietal | Cortex + WM | Present | High | Iso | None | Dilated VR spaces | Yes |

**Supplementary Table 1**. Detailed radiological characteristics of included patients

NA: not available; T1w: T1-weighted; T2w: T2-weighted; VR: Virchow-Robin; WM: white matter.

T1 and T2 weighted signal intensity describes the signal of the involved cortex, and is defined by comparison with normal cortex (high/intermediate/low).
