# Supplementary material for: A comprehensive histomolecular characterization of meningioangiomatosis: Further evidence for a precursor neoplastic lesion
Source: Brain Pathol. 2024 Apr 2;34(6):e13259. doi: 10.1111/bpa.13259 (PMC11483523; doi:10.1111/bpa.13259)
Supplement: Supplementary file 4 — Data S1: Supporting Information [file BPA-34-e13259-s004.docx]

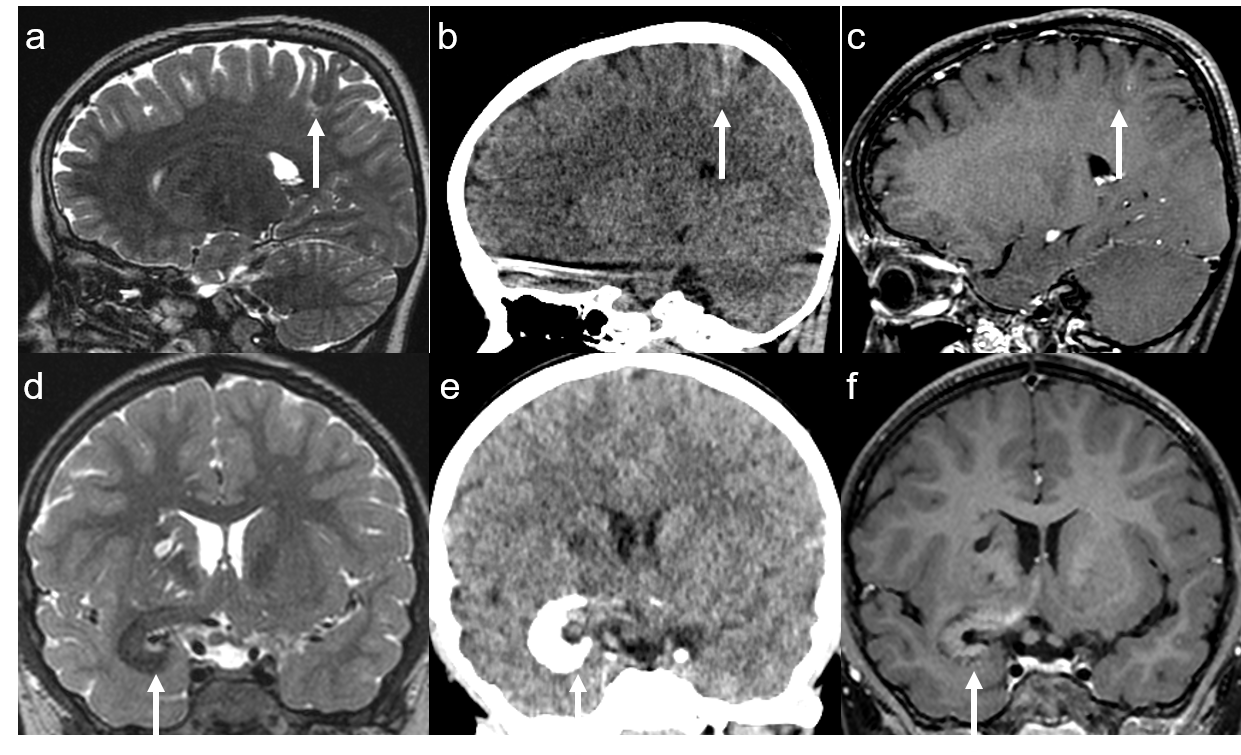


Neuroradiological features of meningioangiomatoses. Magnetic Resonance Imaging of patient #4, showing a cortical T2-weighted low intensity signal with a subcortical high intensity signal (**a**), high density on computed tomography with microcalcification (**b**) and very slight contrast enhancement (**c**). Magnetic Resonance Imaging of patient #1, depicts a thick cortex with T2-weighted low intensity signal and enlarged Virchow-Robin spaces in the basal ganglia (**d**), coarse calcification on computed tomography (**e**) and contrast enhancement (**f**).


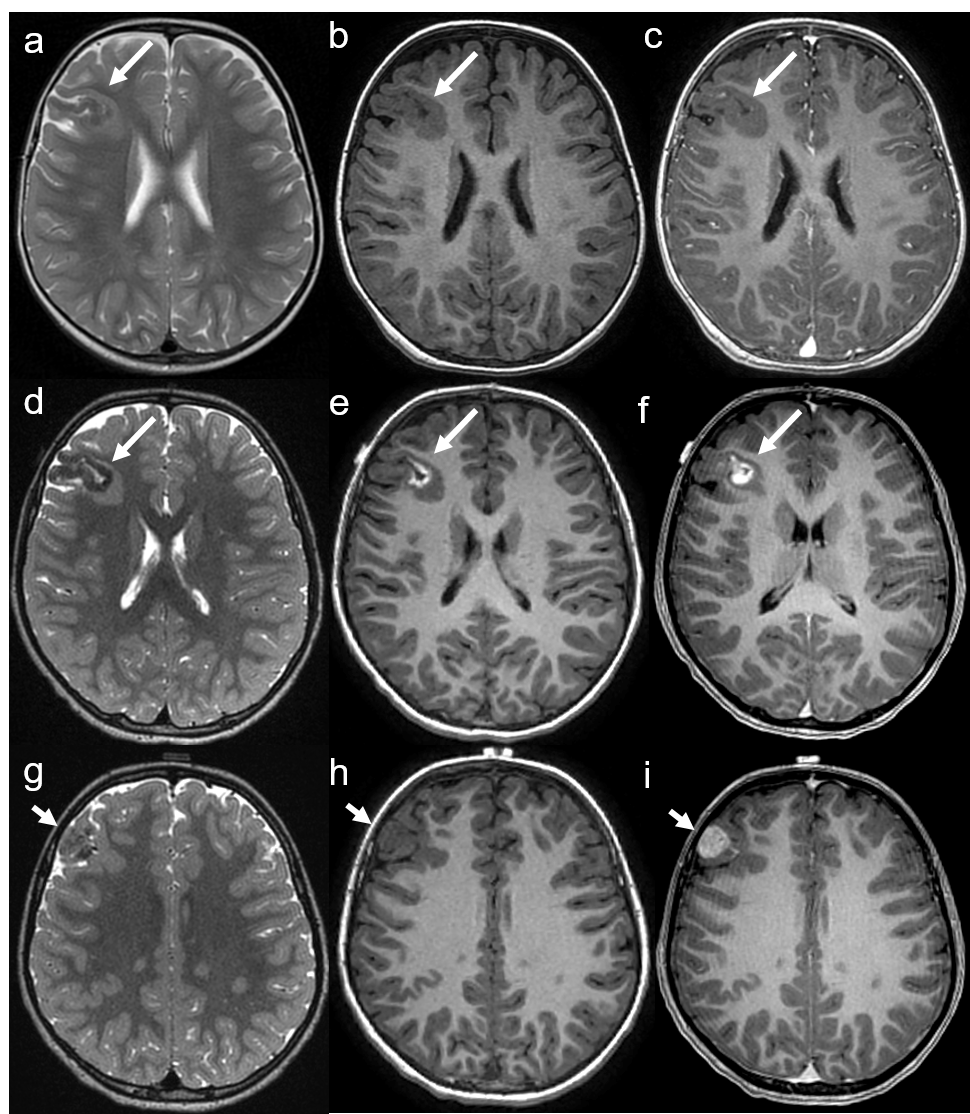


Neuroradiological features of case #9. Magnetic Resonance Imaging performed at diagnosis at the age of 2 (**a-c**) and before surgery at the age of 7 (**d-i**). The right frontal meningioangiomatosis is observed as cortical thickening with T2-weighted low intensity signal (**a**, **d**), an intermediate signal on T1-weighted images at diagnosis (**b**), but high intensity signal during follow-up (**d**), consistent with calcification on computed tomography (not shown), and very small contrast enhancement (**f**). Additionally, a small subcortical T2-weighted high intensity signal is present (**d**). An extra-axial mass with T2 and T1-weighted low intensity signal (**g**, **h**) and strong contrast enhancement (**i**) is indicative of an associated meningioma. It was not visible on the initial Magnetic Resonance Imaging scan (not shown).

**Table 1. Summary of clinical, histopathological and molecular findings**

| **Case** | **Age** | **Sex** | **Histopathological diagnosis (grade)** | **Symptoms** | **Location** | **Medical history** | **DNA-methylation profiling v12.5 (calibrated score)** | **CNV/FISH analyses** | **DNA-sequencing analyses** |
| --- | --- | --- | --- | --- | --- | --- | --- | --- | --- |
| 1 | 9 | M | MAM | Epilepsy | Right temporal | 0 | Ganglioglioma (0.19) | Flat | *KMT2C* |
| 2 | 28 | F | MAM | Epilepsy | Left temporal | 0 | Control tissue (0.12) | Flat | WT |
| 3 | 10 | M | MAM | Epilepsy | Left frontal | 0 | Glioblastoma, IDH-wildtype (0.09) | Flat | WT |
| 4 | 10 | M | MAM | Epilepsy | Parietal | 0 | Desmoplastic infantile ganglioglioma/astrocytoma (0.16) | Flat | WT |
| 5 | 9 | M | MAM | Epilepsy | Left temporal | 0 | Desmoplastic infantile ganglioglioma/astrocytoma (0.14) | Hemizygous del. 22q, del. 13 | WT |
| 6 | 9 | F | MAM | Epilepsy | Right frontal | 0 | Pilocytic astrocytoma, hemispheric (0.18) | Gain 1q | WT |
| 7 | 15 | F | MAM | Fortuitous | Left frontal | Medulloblastoma | Meningioma, subtype benign, subclass 3 (0.30) | Hemizygous del. 22q, del. 1q | WT |
| 8 | 11 | F | Meningothelial meningioma (1) | Epilepsy | Left frontal | 0 | Meningioma, subtype benign, subclass 3 (0.09) | Hemizygous del. 22q, gain 3p | WT |
|  | 11 | F | MAM | Epilepsy | Left frontal | 0 | Desmoplastic infantile ganglioglioma/astrocytoma (0.21) | Hemizygous del. 22q, gain 3p | WT |
| 9 | 7 | M | Atypical meningioma (2) | Epilepsy | Right frontal | 0 | Meningioma, subtype benign, subclass 1 (0.60) | Hemizygous del. 22q, del. 1q, del. 17p | WT |
|  | 7 | M | MAM | Epilepsy | Right frontal | 0 | Desmoplastic infantile ganglioglioma/astrocytoma (0.13) | Hemizygous del. 22q, del. 1q, del. 17p | WT |
| 10 | 12 | M | Atypical meningioma (2) | Epilepsy | Convexity | 0 | Meningioma, subtype benign, subclass 3 (0.30) | Hemizygous del. 22q, *EGFR* amplification, gain 5p | WT |
|  | 12 | M | MAM | Epilepsy | Convexity | 0 | Supratentorial ependymoma, ZFTA fusion-positive (0.08) | Hemizygous del. 22q | WT |
| 11 | 1 | M | Atypical meningioma (2) | Epilepsy | Left parietal | AT/RT | Meningioma, subtype benign, subclass 3 (0.99) | Hemizygous del. 22q, del. 1p, del. 2p | WT |
|  | 1 | M | MAM | Epilepsy | Left parietal | AT/RT | Teratoma (0.33) | Hemizygous del. 22q, del. 2p | WT |

| case | Lobe | Involved structures | Calcifications | T1w signal | T2w  signal | Contrast enhancement | Cysts | Meningioma |
| --- | --- | --- | --- | --- | --- | --- | --- | --- |
| 1 | Temporal | Cortex + WM | Macro | Iso | Low | Moderate | Dilated VR spaces | No |
| 2 | Temporal | Cortex | NA | High | NA | NA | No | No |
| 3 | Frontal | Cortex + WM | NA | Iso | Low | None | Dilated VR spaces | No |
| 4 | Parietal | Cortex + WM | Micro | High | Low | Faint | No | No |
| 5 | Temporal | Cortex + WM | Micro | High | Low | Faint | No | No |
| 6 | Frontal | Cortex + WM | Macro | High | Low | Strong | No | No |
| 7 | Frontal | Cortex | NA | Low | High | None | No | No |
| 8 | Frontal | Cortex + WM | Macro | Iso | Low | High | No | Yes |
| 9 | Frontal | Cortex + WM | Macro | High | Low | Faint | No | Yes |
| 10 | Frontal | NA | NA | NA | NA | NA | NA | NA |
| 11 | Parietal | Cortex + WM | Macro | High | Iso | None | Dilated VR spaces | Yes |

**Supplementary Table 1**. Radiological characteristics of included patients

WM: white matter. T1w: T1-weighted. T2w: T2-weighted. T1 and T2 weighted signal intensity describes the signal of the involved cortex, and is defined by comparison with normal cortex (high/intermediate/low).
